# Pyroptosis Regulators and Tumor Microenvironment Infiltration Characterization in Clear Cell Renal Cell Carcinoma

**DOI:** 10.3389/fonc.2021.774279

**Published:** 2022-01-05

**Authors:** Xi Zhang, Xiyi Wei, Yichun Wang, Shuai Wang, Chengjian Ji, Liangyu Yao, Ninghong Song

**Affiliations:** ^1^ The State Key Lab of Reproductive Medicine, Department of Urology, The First Affiliated Hospital of Nanjing Medical University, Nanjing, China; ^2^ Department of Urology, The Affiliated Kezhou People’s Hospital of Nanjing Medical University, Kezhou, China

**Keywords:** pyroptosis, pyroptosis index, clear cell renal cell carcinoma, *AIM2*, immune microenvironment

## Abstract

**Background:**

It is well known that chronic inflammation can promote the occurrence and progression of cancer. As a type of proinflammatory death, pyroptosis can recast a suitable microenvironment to promote tumor growth. However, the potential role of pyroptosis in clear cell renal cell carcinoma (ccRCC) remains unclear.

**Methods:**

The transcriptome expression profile and mutation profile data of ccRCC with clinical characteristics included in this study were obtained from The Cancer Genome Atlas (TCGA) and Gene Expression Omnibus (GEO) databases. Consensus clustering was used for clustering. Gene set enrichment analysis (GSEA) analysis were applied to evaluate the biological mechanisms. Single sample gene set enrichment analysis (ssGSEA) was applied for evaluating the proportion of various immune infiltrating cells. The ESTIMATE algorithm was involved to compute the immune microenvironment scores.

**Results:**

Among the 17 pyroptosis regulators, a total of 15 pyroptosis regulators were differential expressed between tumor and normal tissues, in which 12 of them emerged strong correlations with prognoses. According to the pyroptosis components, the ccRCC patients were divided into four pyroptosis subtypes with different clinical, molecular, and pathway characteristics. Compared with other clusters, cluster B showed the pyroptosis heat phenotype, while cluster D represented the pyroptosis cold phenotype with poor overall survival. In addition, we performed principal component analysis (PCA) on the differential genes between clusters to construct the pyroptosis index. Furthermore, the pyroptosis index was significantly correlated with survival in different tumor mutation statuses and different grades and stages. Besides, the expression of pyroptosis-related regulators was related to the infiltration of immune cells and the expression of immune checkpoints, among which *AIM2* was considered as the most significant immune-related pyroptosis regulator. Ultimately, we found that *AIM2* was related to the immune activation pathway and was significantly overexpressed in tumor tissues.

**Conclusion:**

This study revealed that pyroptosis regulators and pyroptosis index played an important role in the development and prognoses of ccRCC. Moreover, *AIM2* can be used as a predictor of the response of immunotherapy. Assessing the pyroptosis patterns may help evaluate the tumor status and guide immunotherapy strategies.

## Introduction

As a frequent malignant tumor of the urinary system, renal cell carcinoma (RCC) originated from the renal tubular epithelial system, which accounted for 80% to 90% of the malignant renal tumors (1, 2). RCC accounted for about 2% of all cancer diagnoses and cancer deaths in the world, and about 295,000 new cases of RCC were diagnosed worldwide every year, with about 134,000 deaths recorded (3). Histologically, clear cell renal cell carcinoma (ccRCC) is the prominent subtype of RCC, accounting for approximately 75% of RCC cases (4). Currently, early resection is considered the basic treatment for ccRCC patients, but nearly 30% of local ccRCC patients had recurrence and metastasis after tumor resection (5). Besides, its curative effects remained inadequate for these terminal ccRCC patients (6). Although great progress had been made in screening, diagnosis, surgery, and various treatments, the clinical results of advanced ccRCC were still unsatisfactory (6). Therefore, in order to provide better treatment for ccRCC patients, it was necessary to comprehensively understand the biological mechanism of ccRCC development.

Recently, the role of pyroptosis in cancer growth, invasion, and metastasis has been given more and more attention, and pyroptosis has gradually become a good opportunity to improve the efficacy of cancer immunotherapy (7, 8). Besides, pyroptosis is a newly discovered programmed death mode of inflammatory cells (9). It mainly mediated the activation of a variety of caspases including caspase-1 through inflammasomes, bringing about the ceaseless extension of cells until the break of the cell membrane, which led to the release of cell substance and after that enacted a solid inflammatory response (9, 10).

The aims of the research were to investigate the mechanism of pyroptosis-related regulators (PRRs) in ccRCC and to explore the effect of PRRs in the prognoses of ccRCC patients. We divided ccRCC samples into four pyroptosis clusters and constructed the pyroptosis index (PI) based on the diverse genes between different clusters to predict the survival rate and progress of ccRCC. In addition, we analyzed the correlation of PRRs with the infiltration of immune cells and the expression of immune checkpoints, which may help guide ccRCC immunotherapy strategies.

## Methods

### Clear Cell Renal Cell Carcinoma Dataset Source and Preprocessing

In this study, a total of two eligible ccRCC cohorts [GSE29609 and The Cancer Genome Atlas-Kidney renal clear cell carcinoma (TCGA-KIRC)] were collected for further analysis. Public gene expression data and complete clinical annotations of ccRCC in the GEO and TCGA databases were obtained for analysis, among which cases without survival information and survival status were excluded. Besides, we downloaded somatic mutation data from the TCGA database. R (version 4.1.1) and R Bioconductor package were utilized for analysis. The Wilcoxon test was used to identify differentially expressed PRRs. At the same time, |LogFC| >2 and *P <*0.05 were used as the criteria for screening differentially expressed PRRs. Real-time quantitative PCR (RT-qPCR) was utilized to assess the *AIM2* expression in ccRCC (**Table S1**).

### Establishment of Pyroptosis Clusters

Based on the expression of 17 PRRs, we applied unsupervised cluster analysis to identify disparate PRR modification patterns and classified patients for further analysis. The number of clusters and their steadiness was decided by the consensus clustering calculation. We utilized the ConsensusClusterPlus package to perform the procedures and repeated it 1,000 times to ensure the stability of the classification (11). The overall survival (OS) for each pyroptosis cluster was calculated by employing the Kaplan Meier (KM) curve. Meanwhile, the log-rank test was utilized to evaluate the distinction in survival between the high-expression group and low-expression group with a criteria level of *P <*0.05.

### Identification of the Immune Characteristics of Clusters

In order to further explore the role of PRRs in biological pathway, we utilized the “GSVA” R package to perform gene set variation analysis (GSVA). In non-parametric and unsupervised strategies, GSVA was as a rule utilized to assess changes in pathways and biological process movement in expression dataset tests. We downloaded the gene set of “c2.cp.kegg.v7.4.symbols” from the MSigDB database for running GSVA. A balanced *P*-value of less than 0.05 was considered statistically critical. The ssGSEA score was used to quantify the enrichment level of 23 immune signatures in each KIRC sample. Charoentong’s research had stored various human immune cell subtypes, including activated CD8 T cells, activated dendritic cells, and regulatory T cells. Therefore, we obtained the genome of each TME-infiltrating immune cell type from this study. The relative abundance of each TME-infiltrated cell in each sample was represented by the enrichment score calculated by ssGSEA. In addition, we further analyzed the difference of each cluster and used the VennDiagram package to draw the Venn diagram of the genes that overlap between each cluster. To further confirm the potential functions of the intersection genes, the data were analyzed through function enrichment. Gene Ontology (GO) is a widely used tool for annotating functional genes, especially molecular functions (MFs), biological pathways (BPs), and cellular components (CCs). The Kyoto Encyclopedia of Genes and Genomes (KEGG) enrichment analysis is practical and can be used to analyze gene function and related advanced genome function information. To comprehend the carcinogenic impacts of target genes, the clusterProfiler package was utilized to analyze the GO functions of the overlapping genes and enhance the KEGG pathway. Besides, we also used GSEA to determine the potential molecular mechanism of *AIM2* in KIRC.

### Establishment of the Pyroptosis Index

To measure the PRRs alteration of cancer, we built a set of the scoring framework to assess the PRR adjustment of patients with ccRCC—the pyroptosis index (PI). The strategies established by PI mainly included the following points: the different expression PRRs recognized from different clusters were firstly normalized among all ACRG (Asian Cancer Research Group) tests and the overlapping genes were extricated. At that point, we performed the prognostic analysis for each gene within the signature utilizing the univariate Cox regression model. The genes with a critical prognosis were extricated for advanced investigation. Both central components 1 and 2 were chosen to act as signature scores. This strategy had the advantage of centering the score on the set with the largest block of well-connected (or anticorrelated) qualities within the set; while down-weighting contributions from genes that do not track with other set members. We at that point characterize the index employing a strategy comparable to GGI (12, 13):


$$\text{Index}=\sum (\text{PC}1_{i}+\text{PC}2_{i})$$

Where i represents the expression of PRRs.

### Correlation Between Pyroptosis Index and Other Related Biological Processes

According to the correlation between PI and patient survival rate, the survival R package was utilized to decide the cutoff point of each dataset subgroup. The work of the surv-cutpoint was to more than once test all potential cutoff points to discover the biggest rank measurement so that patients were separated into high and low PI groups based on the biggest chosen log-rank measurement to diminish the calculated batch effect. We used the ggalluvial R package to draw an alluvial map to show the changes between cluster, PI, and survival status. We performed correlation analysis on the PI to further reveal the connection between the PI and immune cells. The maftools function was used to present a waterfall chart of the mutation landscape of patients with high and low PI clusters. The KM method was used to analyze the survival correlation between the high and low PI groups and clinical grouping information. Finally, we downloaded the scoring data of 537 cases of KIRC immunotherapy from The Cancer Immunome Database (TCIA) and analyzed the correlation between high- and low-risk groups and immunotherapy. All statistical *P*-values were two-sided, with *P <*0.05 as statistically critical. All data preparation was completed by R 4.1.1 software.

## Results

### Genetic Variation Prognoses of Pyroptosis Regulators in Clear Cell Renal Cell Carcinoma

**Figure 1:**
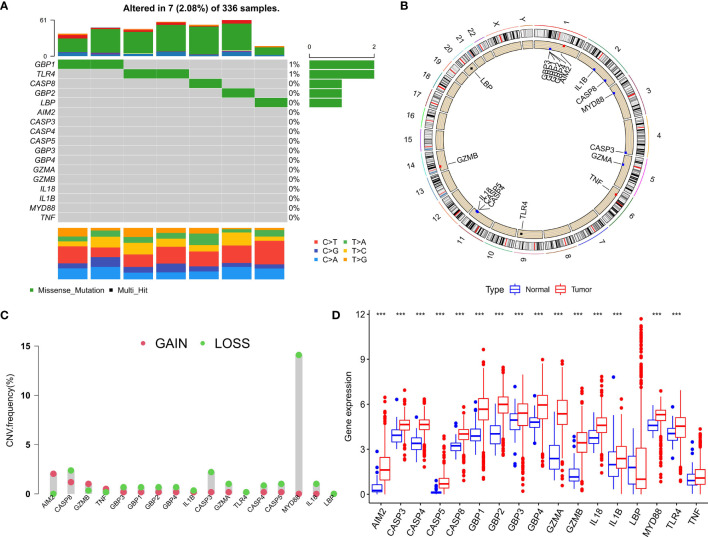
Expression and genetic variation of pyroptosis regulators in clear cell renal cell carcinoma. **(A)** The mutation frequency of 17 pyroptosis regulatory factors for 336 ccRCC patients in the TCGA-KIRC cohort. **(B)** The location of CNV alteration of pyroptosis regulators on 23 chromosomes. **(C)** The CNV variation frequency of pyroptosis regulators. red circle: amplified frequency; green circle: missing frequency. **(D)** The expression difference of 17 pyroptosis regulatory factors between normal tissue and ccRCC tissue. The asterisk represents the statistical p value (*P < 0.05; **P < 0.01; ***P < 0.001).

**Figure 2:**
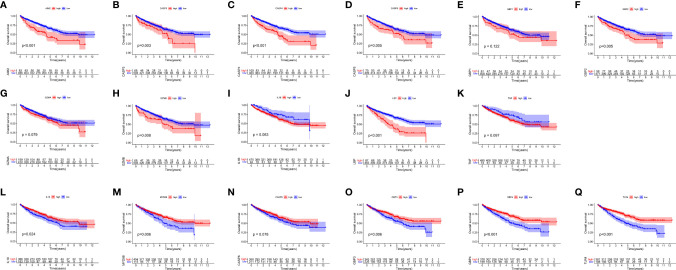
The OS Kaplan-Meier curve of 17 pyroptosis regulators in the TCGA-KIRC. **(A–K)** 11 pyroptosis regulators were negatively correlated with survival. **(L–Q)** 6 pyroptosis regulators were positively correlated with survival.

After merging the GEO and TCGA databases, we finally identified 17 PRRs. Among the 336 samples, only 7 samples had PRR mutations, and the mutation rate was 2.08%. It was found that only *GBP1*, *CASP8*, *TLR4*, *GBP2*, and *LBP* had mutations, and none of the other genes showed any mutations in the KIRC samples (**Figure 1A**). **Figure 1B** showed the copy number variation (CNV) changes of the PRRs on the chromosome. In addition, through the analysis of the frequency of CNV changes, it was found that CNV changes were common in 17 genes and most of them were concentrated in the loss of copy number, while the loss of MYD88 was the most obvious. In addition, only the copy number of *AIM2* was amplified (**Figure 1C**). To determine whether the abovementioned genetic variation affected the expression of PRRs in ccRCC patients, we further analyzed the expression levels of PRRs in normal and tumor samples. It was discovered that the genes other than *TNF* and *LBP* were significantly separate between normal and tumor samples (**Figure 1D**). However, compared with normal tissues, the expression of PRRs deleted in CNV was significantly higher in tumor tissues (**Figures 1C, D**). The above results showed that although the expression of PRRs was highly heterogeneous in normal and tumor samples, the alteration of CNV may not be the main factor causing the perturbation of PRRs. To clarify the impacts of these 17 PRRs on the prognoses of ccRCC, we carried out the survival analysis. The results showed that a total of 12 PRRs have significant differences, of which 7 PRRs were negatively correlated with prognoses and the rest were positively correlated. In addition, 5 genes were not different between tumor and normal tissues (**Figure 2**).

### Identification of the Immune Characteristics and Prognoses of Each Pyroptosis Cluster

**Figure 3:**
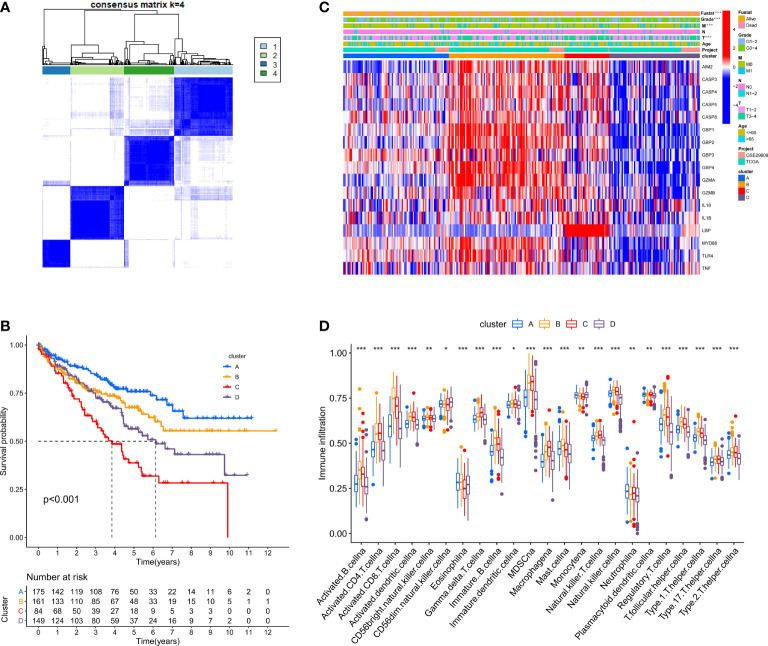
Identification of potential pyroptosis clusters in ccRCC patients. **(A)** Sample distribution of different clusters. **(B)** The OS Kaplan-Meier curve of different clusters in ccRCC patients. **(C)** The heatmap showing gene expression and clinical correlation among different clusters of ccRCC. **(D)** Differential expression of immune cells among the KIRC pyroptosis clusters. The asterisk represents the statistical p value (*P < 0.05; **P < 0.01; ***P < 0.001).

We used the ConsensusClusterPlus package to classify patients depending on the expression levels of 17 PRRs and finally distinguished 4 diverse clusters (**Figure 3A**). Next, survival analysis was conducted based on the four clusters. The results showed that there was critical statistical diversity between the clusters, and cluster A had the most significant survival advantage (**Figure 3B**). In addition, the correlation heat map indicated the expression of PRRs and various clinical information including survival status, grade, TMN, and age among clusters A–D. The outcomes showed that the expression of PRRs was generally higher in B and C (**Figure 3C**). Besides, to explore the immune atlas of pyroptosis clusters, we first investigated the layout of 23 immune cells in each cluster. The results showed that innate immune cell infiltration was significantly enriched in cluster B including activated CD8 T cell, immature B cell, natural killer cell, MDSC, mast cell, T follicular helper cell, and type 1 T helper cell (**Figure 3D**). In addition, patients in cluster B showed better survival advantage (**Figure 3B**). However, although the degree of innate immune cell infiltration was low in cluster A, it had an obvious survival advantage (**Figures 3B, D**).

### Identification of GSVA and Functional Enrichment Analysis Between Pyroptosis Clusters

**Figure 4:**
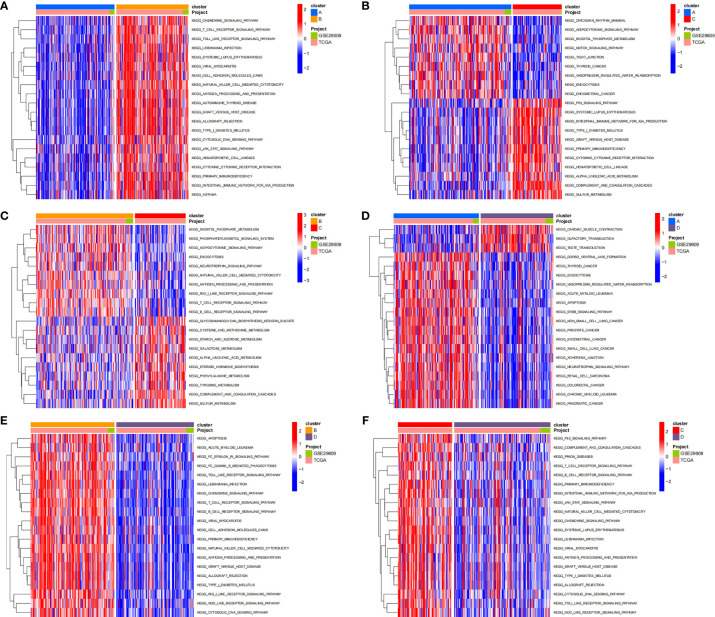
The results of GSVA enrichment analysis. The heatmap showing the results of GSVA enrichment analysis among different pyroptosis clusters. Red represented activated pathways; blue represented inhibited pathways. (**A**: cluster A vs cluster B; **B**: cluster A vs cluster C; **C**: cluster B vs cluster C; **D**: cluster A vs cluster D; **E**: cluster B vs cluster D; **F**: cluster C vs cluster D).

**Figure 5:**
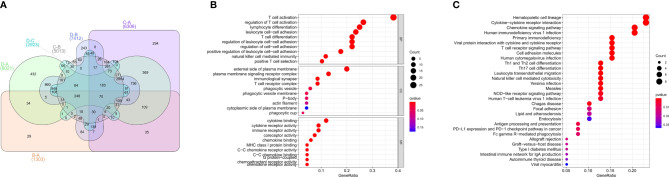
GO, KEGG analysis of intersective differential genes between clusters. **(A)** Venn diagram of 70 intersection genes. **(B)** Bubble of GO enrichment analysis results for 70 intersection genes. **(C)** Bubble of KEGG enrichment analysis results for 70 intersection genes.

To investigate the biological behaviors of these PRRs, we performed GSVA. Cluster A showed obvious immunosuppression, where adipocytokine_SIGNALING_PATHWAY, INOSITOL_PHOSPHATE_Metabolism, and notch_SIGNALING_Pathway were significantly enriched (**Figures 4A, B**). Cluster B showed enrichment pathways related to complete immune activation, including CHEMOKINE_SIGNALING_PATHWAY, T_CELL_RECEPTOR_SIGNALING_PATHWAY, and toll_LIKE_RECEPTOR_SIGNALING_ PATHWAY (**Figure 4A**). Besides, the P53 SIGNALING PATHWAY, JAK–STAT SIGNALING PATHWAY, and CHEMOKINE SIGNALING PATHWAY were significantly enriched in cluster C (**Figure 4F**). Similarly, cluster D showed obvious immunosuppression (**Figure 4E**). To our surprise, we found that cluster C was significantly enriched in innate immune cell infiltration, including natural killer cells, macrophages, CD4-positive T cells, and MDSC cells. However, patients with this PRR modification pattern showed the worst survival advantage. We speculated that this may be related to the abnormal activation of the JAK–STAT signaling pathway and p53 signaling pathway. Then, we screened the differential genes between the two clusters according to the adjusted *P <*0.001 standards. Then, the differential genes in these 6 groups (A–B, A–C, A–D, B–C, B–D, C–D) were crossed, and finally, 70 crossed genes were obtained (**Figure 5A**). The selected 70 intersection genes were further analyzed by GO annotation and KEGG enrichment. The results of BP showed that the 70 intersection genes were mainly concentrated in T-cell activation, regulation of T-cell activation, lymphocyte differentiation, T-cell differentiation, leukocyte cell–cell adhesion, and regulation of leukocyte cell–cell adhesion. Regarding MFs, the target genes were usually enriched in cytokine binding, cytokine receptor activity, and immune receptor activity. Regarding CCs, target genes were mainly concentrated on the external side of the plasma membrane, plasma membrane signaling receptor complex, and immunological synapse (**Figure 5B**). KEGG annotation results demonstrated enrichment in hematopoietic cell lineage, cytokine–cytokine receptor interaction, chemokine signaling pathway, and human immunodeficiency virus 1 infection (**Figure 5C**).

### Identification of the Tumor Mutation Burden and Clinical Characteristics of the Pyroptosis Index

**Figure 6:**
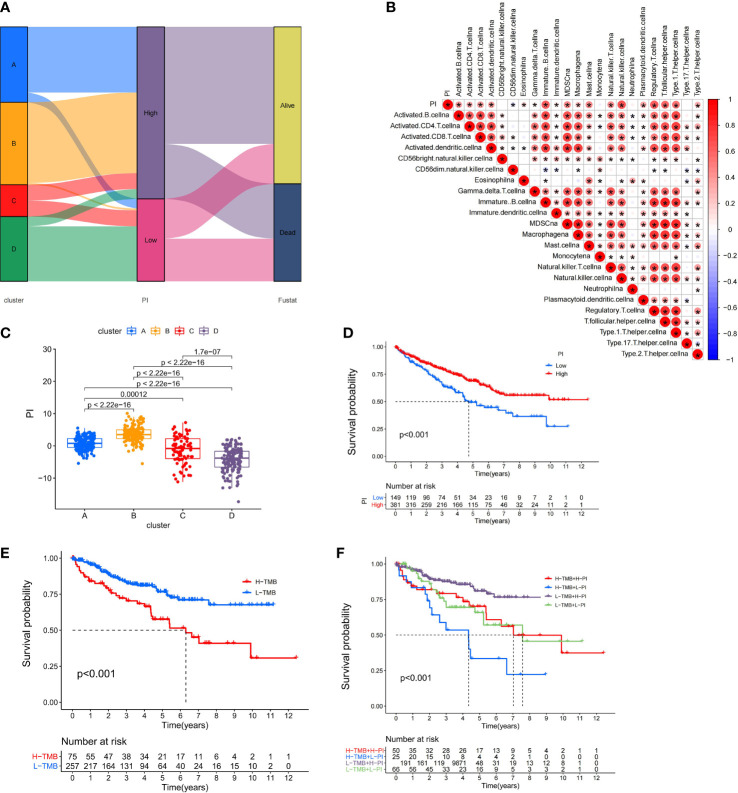
Immunological and tumor somatic mutation characteristics of pyroptosis index. **(A)** Alluvial diagram showing the changes of clusters, index, and fustat. **(B)** Correlations between the PI and immune cells. **(C)** Differential expression of PI among the KIRC pyroptosis clusters. **(D)** The OS Kaplan-Meier curve of High and Low PI. **(E)** The OS Kaplan-Meier curve of high and low TMB groups. **(F)** The OS Kaplan-Meier curve of the combination of PI and TMB.

**Figure 7:**
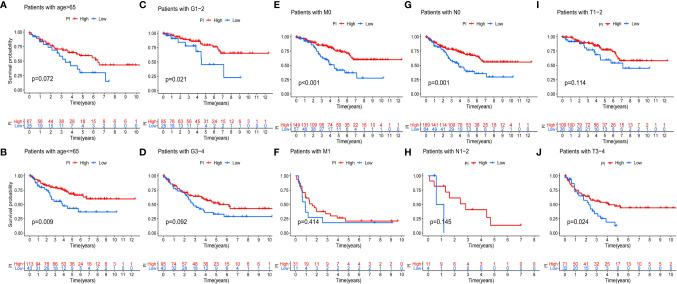
Validation of the prognosis of PI under the stratifications of different clinical parameters. **(A, B)** age > 65 and age <=65; **(C, D)** histological grade 1-2 and 3-4; **(E, F)** M stage 0 and 1 **(G, H)** N stage 0 and 1; **(I, J)** T stage 1-2 and 3-4.

Because of the heterogeneity and complexity of PRR modification, we constructed the PI to evaluate the PRR modification pattern of individual patients. The alluvial map was used to visualize the attribute changes of a single patient (**Figure 6A**). To more usefully outline the characteristics of the PI, we, moreover, analyzed the relationship between immune cells and PI. The outcomes appeared that the index was closely associated with immature B cell, natural killer cell, regulatory T cell, and T follicular helper cell (**Figure 6B**). The results of the Kruskal–Wallis test revealed that compared with other clusters, cluster B showed a significantly increased index (**Figure 6C**). Moreover, the results showed that the index was positively related to the patient prognoses (**Figure 6D**). There was no doubt that the L-TMB had a better survival advantage (**Figure 6E**). Survival analysis combining mutation and index proved the accuracy of the above results (**Figure 6F**). In order to explore whether the index applied to patients of various clinical groups, we used the KM curve to analyze whether there were prognosis contrasts between high and low PI groups among diverse clinical groups. The results showed that there were critical statistical differences between high index and low index in age ≤65, G1–2, M0, N0, and T3–4 groups. Compared with the low-index group, the high-index group had a significant survival advantage (**Figure 7**). Then, the maftools package was employed to investigate the discrepancy of somatic mutations between the low-index group and the high-index group in the TCGA-KIRC cohort. **Figures S1A, B** reveal that the high-index group presented broader tumor mutation burden than the low-index group. In addition, we investigated the relationship between the PI and survival status. The consequence of the histogram and block diagram indicated that the high-index group had better survival advantages (**Figures S1C, D**). Besides, we tested whether PI can be used as an independent prognostic biomarker for ccRCC. Therefore, we constructed a nomogram to speculate the prognoses of KIRC (**Figure S2A**). Meanwhile, multivariate Cox regression analysis confirmed that PI was a credible and independent prognostic biomarker for assessing the prognoses of ccRCC patients (HR = 0.945; 0.901−0.991) (**Figures S2B, C**). In particular, we inspected the capacity of PI to predict the curative effect of immunotherapy in ccRCC patients. Besides, anti-PD-1 therapy was more suitable for high-index patients (**Figure S2D**), while anti-CTLA-4 therapy response was not different between the high- and low-index groups (**Figure S2E**).

### Identification of the Immune Characteristics of Pyroptosis Regulators

**Figure 8:**
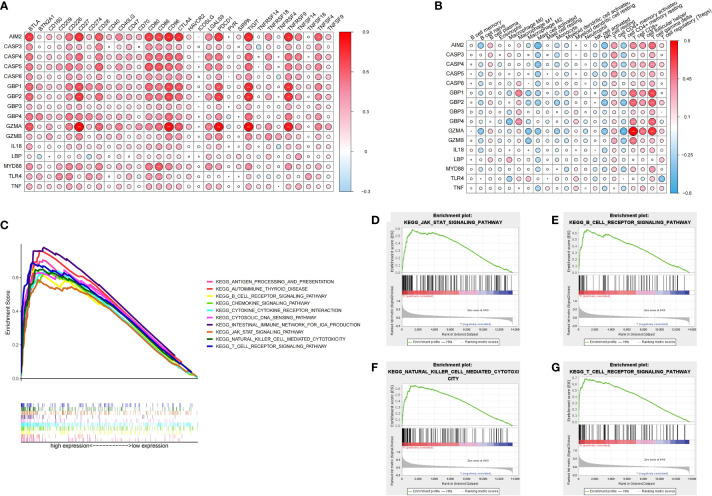
Immune characteristics and enrichment pathway characteristics of pyroptosis related regulators. **(A)** Association between pyroptosis regulators and immune cells. **(B)** Association between pyroptosis regulators and immune checkpoints. **(C–G)** The results of GSEA analysis of AIM2.

In order to systematically study the potential influence of PRRs on the immune microenvironment of ccRCC, we utilized correlation analysis for PRRs and infiltrating immunocytes and immune checkpoints. The expression level of immune checkpoints was positively related to PRRs (**Figure 8A**). Considering the known role of checkpoints in the immunosuppressive microenvironment, PRRs may have important biological functions in ccRCC immunotherapy. Besides, correlation analysis found that PRRs were negatively associated with various immune cells (**Figure 8B**). Collectively, PRRs were positively correlated with CD8^+^ T cell, follicular helper T cell, and gamma delta T cell and negatively correlated with mast cell, monocyte, and myeloid dendritic cell. In view of the remarkable mutation characteristic, prognostic, and immunoregulatory effects of *AIM2*, GSEA was used to examine *AIM2*-related signaling pathways. GSEA results showed that *AIM2* was involved in multiple malignant pathways, including JAK–STAT SIGNALING PATHWAY, B-CELL RECEPTOR SIGNALING PATHWAY, NATURAL KILLER CELL-MEDIATED CYTOTOXICITY, and T-CELL RECEPTOR SIGNALING PATHWAY (**Figures 8C–G**).

### Validation of *AIM2* in Tissues and Cell Lines

**Figure 9:**
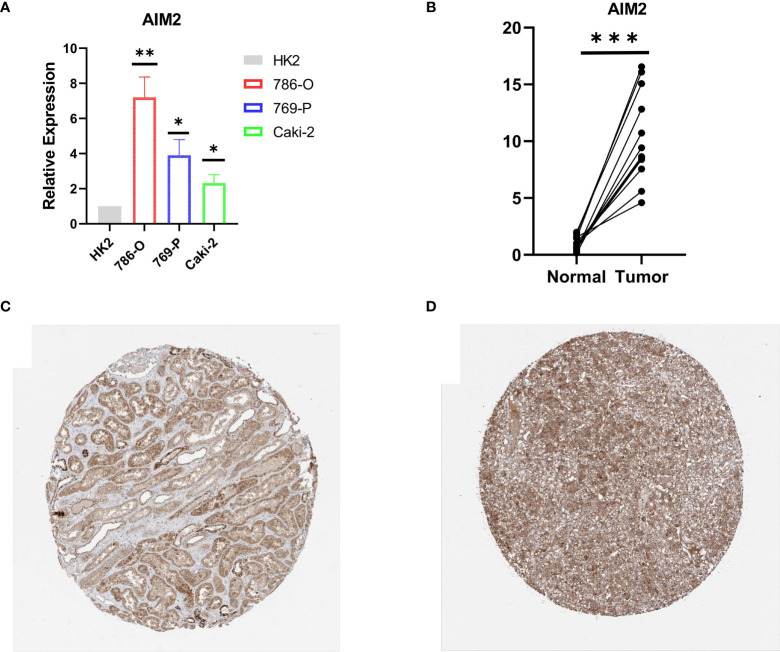
Validation of AIM2 in ccRCC tissues and cell lines. **(A)** Bar plot for the relative expression of AIM2 in ccRCC cell lines and normal HK2 cell line. **(B)** Bar plot for the relative expression of AIM2 between ccRCC tissues and normal tissues. **(C)** Immunohistochemical analysis of AIM2 in normal kidney tissues. **(D)** Immunohistochemical analysis of TRIM2 in ccRCC tissues.

RT-qPCR was carried out in 15 pairs of ccRCC tissues and normal renal tissues and 4 cell lines, consisting of 3 tumor cell lines and 1 normal renal cell line. Compared with normal renal cell lines, the expression of *AIM2* in tumor cell lines was significantly higher, and the expression was the highest in 786-O cell lines (**Figure 9A**). Besides, in tumor tissues, the expression level of *AIM2* was significantly higher than that in normal renal tissues (**Figure 9B**). The above experimental results were consistent with the results predicted by bioinformatics methods. In addition, the Human Protein Atlas (HPA) database explicitly uncovered that in ccRCC tissues, the expression levels of *AIM2* were significantly higher than those in normal renal tissue (**Figures 9C, D**).

## Discussion

In recent years, the morbidity and mortality of RCC have gradually increased, which has become the most fatal adult renal malignancy (14). With the development of scientific research, the traditional histopathological features (tumor size, stage, and grade) may not meet the needs of diagnosis and prognoses (15, 16). Pyroptosis, the lytic programmed cell death, was a pivotal fibrotic mechanism in the development of renal pathology, which has been broadly investigated in inflammatory disease models (17, 18). Recently, more and more shreds of evidence have shown that in the absence of any bacterial or viral infection, pyroptosis can be chemically induced in cancer cells (19, 20). Therefore, we investigated the expression of PRRs in ccRCC and established PI to further predict the role of PRRs in prognoses. In addition, we analyzed the biological function of *AIM2*, which may be an effective immune target.

Through the results, we found that the PRRs played important roles in the development and invasion of ccRCC. The expression of the majority of PRRs in tumor tissues was significantly increased compared with that in normal tissues. Most of the PRRs were critically associated with the prognoses of ccRCC and appeared to be cancer-promoting genes, which led to poor prognoses. With more and more research carried out, scholars found that PRRs performed disparate roles in various cancers (21). For instance, the upregulation of PRR expression in non-small cell lung cancer and breast cancer (BC) was related to poor prognoses (21, 22). On the contrary, many studies have also shown the antitumor activity of the PRRs. The low expression of PRRs in gastric cancer (GC), hepatocellular carcinoma (HCC), and colorectal cancer (CRC) was associated with poor prognoses (23–26).

Here, the KIRC cohort was divided into four clusters *via* the 17 PRRs. Among them, B and C were closely related to PRRs, while cluster A and cluster D were the opposite. In addition, there was more immune cell infiltration in cluster B and cluster C and less in cluster A and cluster D. We speculated that the PRRs had used two signaling pathways (the classical pathway that depended on caspase-1 and the non-classical pathway that depended on caspase-4, 5, and 11) to cleave the precursors of IL-1β and IL-18 to form active IL-1β and IL-18. The active IL-1β and IL-18 were released to the outside of the cell, which recruited immune cells to gather and amplified the inflammatory response (27). Although cluster C was rich in immune cells, it failed to match the corresponding survival advantage. It was possibly because cancer cells may functionally shape their microenvironment by secreting various cytokines, chemokines, and other factors, which led to the reprogramming of the surrounding immune cells. Eventually, a microenvironment that promoted tumor growth and metastasis was formed, which showed the phenomenon of immune evasion (28, 29). The JAK–STAT pathway regulated and controlled tumor cell proliferation, differentiation, and metastasis and played a significant part in regulating the tumor microenvironment (30). The abnormal activation of the JAK–STAT pathway in cluster C promoted the production of some tumor-derived factors such as IL-6, TGF-β, VEGF, and other factors. In addition, it also promoted the recruitment and activation of dendritic cells (DCs), myeloid-derived suppressor cells (MDSCs), tumor-associated macrophages (TAMs), and other cells in the microenvironment. Meanwhile, it released a great quantity of the immunosuppressive factors, recruited the abundance of the immunosuppressive cells, and finally formed a vicious circle of immunosuppressive tumor microenvironment (30). In addition, the JAK/STAT3 pathway may enhance TGF-β-induced epithelial–mesenchymal transition (EMT) to promote tumor metastasis (31).

Considering the individual heterogeneity of genetic modification of pyroptosis, there was an urgent need to quantify the pattern of genetic modification of pyroptosis in individual tumors. To this end, we established the PI to evaluate the PRR modification pattern for individual KIRC patients. We demonstrated that the index can be used to evaluate the prognosis characteristics of patients. Similarly, the PI can be used as an independent prognostic biomarker to predict patient survival. Other than that, we were able to, moreover, foresee the adequacy of the clinical reaction of patients to PD-L1 immunotherapy through the PI. Previously, Khadirnaikar et al. developed and verified an immune lncRNA prognostic score for KIRC patients, which may be used as an independent indicator for judging the prognoses of KIRC patients (32). Diversely, we used PRRs to establish the pyroptosis index for KIRC patients. Besides, Sun et al. constructed a prognostic risk model related to pyroptosis, which can divide KIRC patients into low- and high-risk subgroups with different prognoses and immune cell infiltration. They also systematically analyzed the prognostic value of PRRs in KIRC, their role in TME, their response to ICIs, and drug sensitivity, providing new insights into the role of pyroptosis in KIRC patients in TME and even helping to develop new treatment strategies (33). Compared with us, we firstly systematically and comprehensively analyzed the prognostic characteristics and immune correlation of all PRRs. Secondly, we determined that *AIM2* was the most significant immune-related PRRs in ccRCC, and through *in vitro* experiments, we were able to verify our conclusions.

*AIM2* was confirmed to be closely related to immune activation through GSEA and was positively correlated with immune checkpoints. *AIM2* contained a PYD domain matching the NLRP and a hematopoietic IFN-inducible nucleoprotein with a 200 amino acid repeat (HIN200) domain that recognized exogenous dsDNA. In addition to these two pattern recognition receptors, there were two other proteins, ASC and caspase-1, which were related to the formation of the inflammatory complex. Besides, *AIM2* played a significant part in the development and invasion of tumors and played pro- or anticancerous roles in different tumors. For instance, low expression of *AIM2* in HCC was related to lower OS (34). The reason may be that *AIM2* deficiency enhanced the expression of fibronectin-1 and EMT, thus promoting the metastasis of HCC (34). In addition, *AIM2* may inhibit the growth of HCC by inhibiting the mTOR/S6K1 pathway (35). The high expression of *AIM2* was associated with a higher survival rate in EBV-induced nasopharyngeal carcinoma (NPC). The effect of *AIM2* may be related to IL-1β and immune stimulation of neutrophils to accumulate into tumors to mediate antitumor activity (36). Furthermore, *AIM2* played an protumor role in colorectal tumors because overexpression of *AIM2* in colorectal cancer cells induced the expression of invasion‐associated genes such as *VIM* and *MCAM* (37). *AIM2* was overexpressed in non-small cell lung cancer and facilitated cell proliferation (38, 39). In addition, our research showed that *AIM2* was highly expressed in ccRCC and promoted tumor development through immune activation pathways.

## Conclusion

In a word, our research indicated that PRRs served as critical parts in the development and prognoses of ccRCC. In addition, we established the PI, which can be used to evaluate the clinical, prognostic, and immune patterns. Besides, *AIM2* may regulate the expression of the immune checkpoints, which was a potential immunotherapy target.

## Data Availability Statement

The datasets presented in this study can be found in online repositories. The names of the repository/repositories and accession number(s) can be found in the article/**Supplementary Material**.

## Ethics Statement

The studies involving human participants were reviewed and approved by the Ethical Committee of The First Affiliated Hospital of Nanjing Medical University. The patients/participants provided their written informed consent to participate in this study.

## Author Contributions

NS designed this work. XZ and XW wrote the manuscript. YW performed the bioinformatics analysis. CJ, LY, and SW performed the data review. All authors contributed to the article and approved the submitted version.

## Funding

This work was supported by the National Natural Science Foundation of China (grant number 82071638).

## Conflict of Interest

The authors declare that the research was conducted in the absence of any commercial or financial relationships that could be construed as a potential conflict of interest.

## Publisher’s Note

All claims expressed in this article are solely those of the authors and do not necessarily represent those of their affiliated organizations, or those of the publisher, the editors and the reviewers. Any product that may be evaluated in this article, or claim that may be made by its manufacturer, is not guaranteed or endorsed by the publisher.

## Supplementary Material

Supplementary Figure 1 Characteristics of tumor somatic mutation and survival in high and low index groups. **(A, B)** The waterfall plot of tumor somatic mutation established by high and low PI groups. **(C)** Proportion distribution of fustat in the PI groups. **(D)** Differential expression of PI in fustat of KIRC patients.

Supplementary Figure 2 The role of pyroptosis index in immunotherapy and prognoses evaluation **(A)** The nomogram to predict ccRCC patients. **(B, C)** Univariate and Multivariate Cox regression analysis for PI in KIRC shown by the forest plot. **(D–G)** Differential expression of anti-CTLA4 and anti-PD1 combination immunotherapy among the KIRC PI.
