# Supplementary figures and images for: Pyroptosis Regulators and Tumor Microenvironment Infiltration Characterization in Clear Cell Renal Cell Carcinoma
Source: Front Oncol. 2022 Jan 5;11:774279. doi: 10.3389/fonc.2021.774279 (PMC8766752; doi:10.3389/fonc.2021.774279)

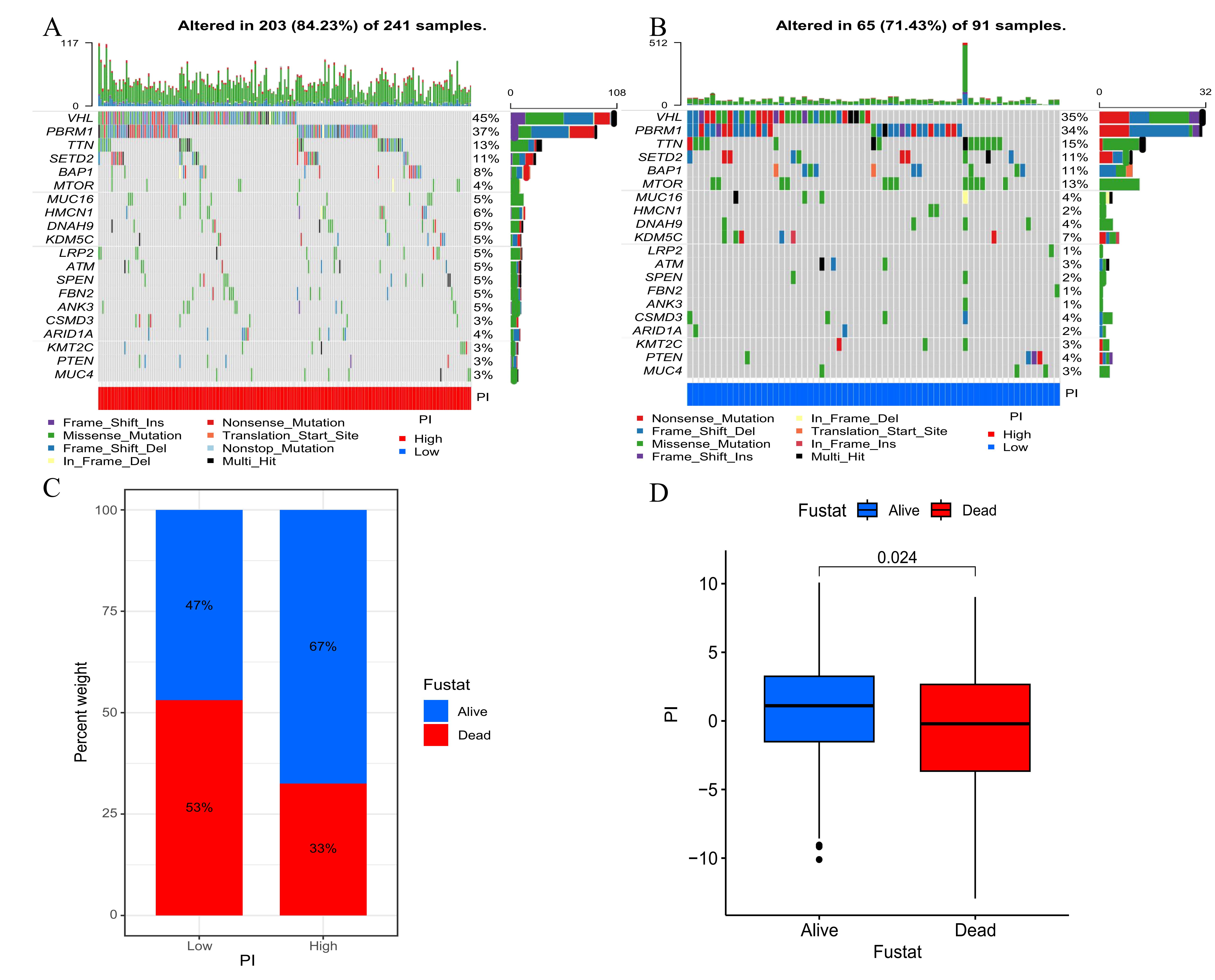

Supplement: Supplementary Figure 1 — Characteristics of tumor somatic mutation and survival in high and low index groups. (A, B) The waterfall plot of tumor somatic mutation established by high and low PI groups. (C) Proportion distribution of fustat in the PI groups. (D) Differential expression of PI in fustat of KIRC patients. [file Image_1.jpg]

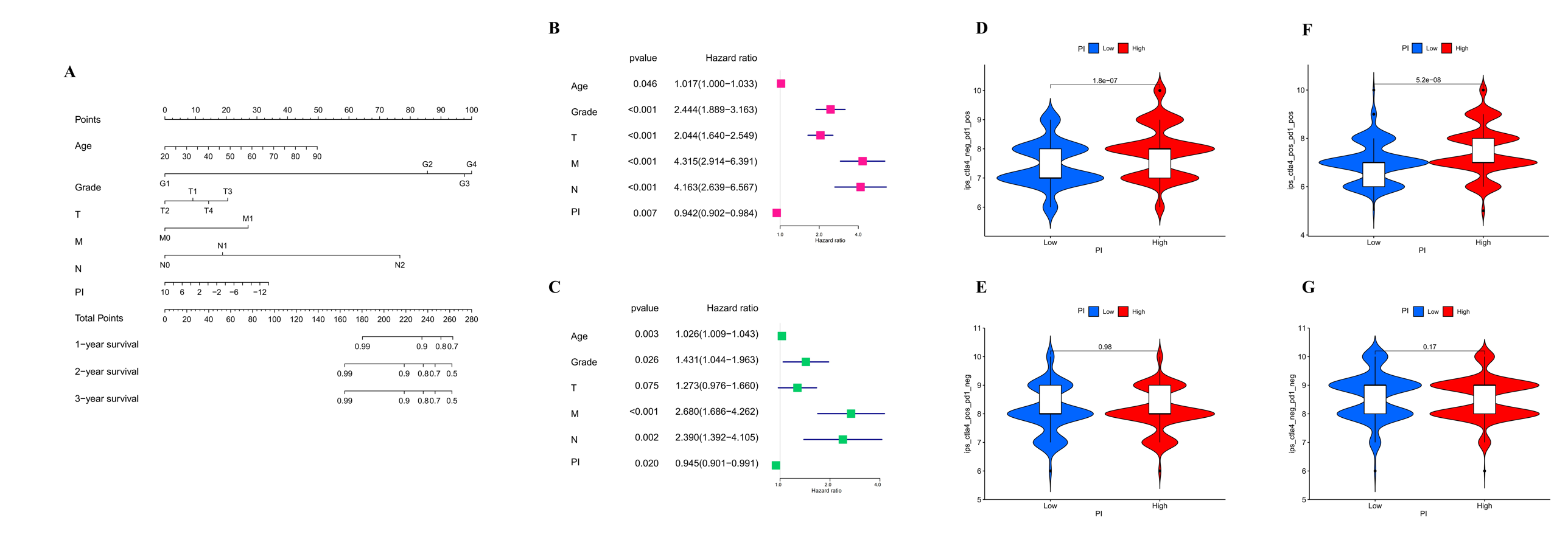

Supplement: Supplementary Figure 2 — The role of pyroptosis index in immunotherapy and prognoses evaluation (A) The nomogram to predict ccRCC patients. (B, C) Univariate and Multivariate Cox regression analysis for PI in KIRC shown by the forest plot. (D–G) Differential expression of anti-CTLA4 and anti-PD1 combination immunotherapy among the KIRC PI. [file Image_2.jpg]
