# Supplementary material for: Pyroptosis Regulators and Tumor Microenvironment Infiltration Characterization in Clear Cell Renal Cell Carcinoma
Source: Front Oncol. 2022 Jan 5;11:774279. doi: 10.3389/fonc.2021.774279 (PMC8766752; doi:10.3389/fonc.2021.774279)
Supplement: Supplementary file 3 [file Table_1.docx]

**Primers of AIM2 and actin**

The primers applied for AIM2 were displayed as follows: forward:

TGGCAAAACGTCTTCAGGAGG; reverse: AGCTTGACTTA

GTGGCTTTGG.

The primers applied for actin were displayed as follows: forward: AGCGAGCATCCCCCAAAGTT; reverse: GGGCACGAAGGCTCATCATT.
